# 3D printing cytoskeletal networks: ROS-induced filament severing leads to surge in actin polymerization

**DOI:** 10.1101/2025.03.19.644260

**Published:** 2025-03-20

**Authors:** Thomas Litschel, Dimitrios Vavylonis, David A. Weitz

**Affiliations:** 1 School of Engineering and Applied Sciences, Harvard University, Cambridge, MA, USA; 2 Department of Physics, Lehigh University, Bethlehem, PA, USA; 3 Department of Physics, Harvard University, Cambridge, MA, USA; 4 Wyss Institute for Biologically Inspired Engineering, Harvard University, Boston, MA, USA

## Abstract

The cytoskeletal protein actin forms a spatially organized biopolymer network that plays a central role in many cellular processes. Actin filaments continuously assemble and disassemble, enabling cells to rapidly reorganize their cytoskeleton. Filament severing accelerates actin turnover, as both polymerization and depolymerization rates depend on the number of free filament ends — which severing increases. Here, we use light to control actin severing in vitro by locally generating reactive oxygen species (ROS) with photosensitive molecules such as fluorophores. We see that ROS sever actin filaments, which increases actin polymerization in our experiments. However, beyond a certain threshold, excessive severing leads to the disassembly of actin networks. Our experimental data is supported by simulations using a kinetic model of actin polymerization, which helps us understand the underlying dynamics. In cells, ROS are known to regulate the actin cytoskeleton, but the molecular mechanisms are poorly understood. Here we show that, in vitro, ROS directly affect actin reorganization.

Actin is the most abundant structural protein in eukaryotic cells, forming a dynamic filament network that undergoes continuous assembly and disassembly.^1^ This turnover allows cells to rapidly reorganize their cytoskeleton in response to environmental and internal signals.

A key constraint on actin assembly is the formation of new filaments de novo, which is a kinetically slow process. Actin polymerization is nucleation-limited, meaning that polymerization via filament elongation is much faster than through the formation of new filaments.^2^ Therefore, the amount of actin polymerization directly correlates with the number of existing filaments.

As nucleation from monomers is slow, filament severing offers an alternative mechanism to increase the number of filaments. When a filament is severed, the number of polymerizing ends doubles. This has the potential to progress exponentially, as these filaments regrow and can undergo further severing. Despite extensive research on actin nucleation, relatively little is known about the mechanisms and consequences of actin severing, particularly regarding its role in actin assembly and disassembly.

Filament severing can occur mechanically, such as through shear stress, motor protein activity, or thermal fluctuations.^3–5^ However, these effects disproportionately affect long filaments. In cells, actin severing can occur through molecular mechanisms such as those mediated by specific protein interactions. For instance, the actin-binding protein cofilin severs filaments, though this severing activity is coupled to cofilin’s other functions.^6^

Another class of compounds, reactive oxygen species (ROS), are intriguing, as they have been shown to greatly affect actin polymerization in vivo.^7–9^ ROS are among the most reactive molecules in biological systems^10,11^ and oxidize and damage molecules including actin monomers.^12^ However, the mechanisms through which ROS affect actin polymerization are not understood. If ROS oxidize monomers within filaments, this could cause filaments to break, increase the number of filaments and affect the dynamics of actin assembly.

Here, we show that ROS interact with actin filaments and can increase actin polymerization drastically in vitro. We describe a simple experimental system that allows for the local generation of ROS via light-excitation of fluorophores. We observe that ROS induce actin fragmentation and effectively increase the number of polymerizing filaments. However, beyond a certain threshold, excessive severing leads to a transition from enhanced polymerization to the disassembly of actin networks. As such, via light, we have control over both actin assembly and disassembly in 3D.

## Localized increase in actin polymerization

**Figure 1.**
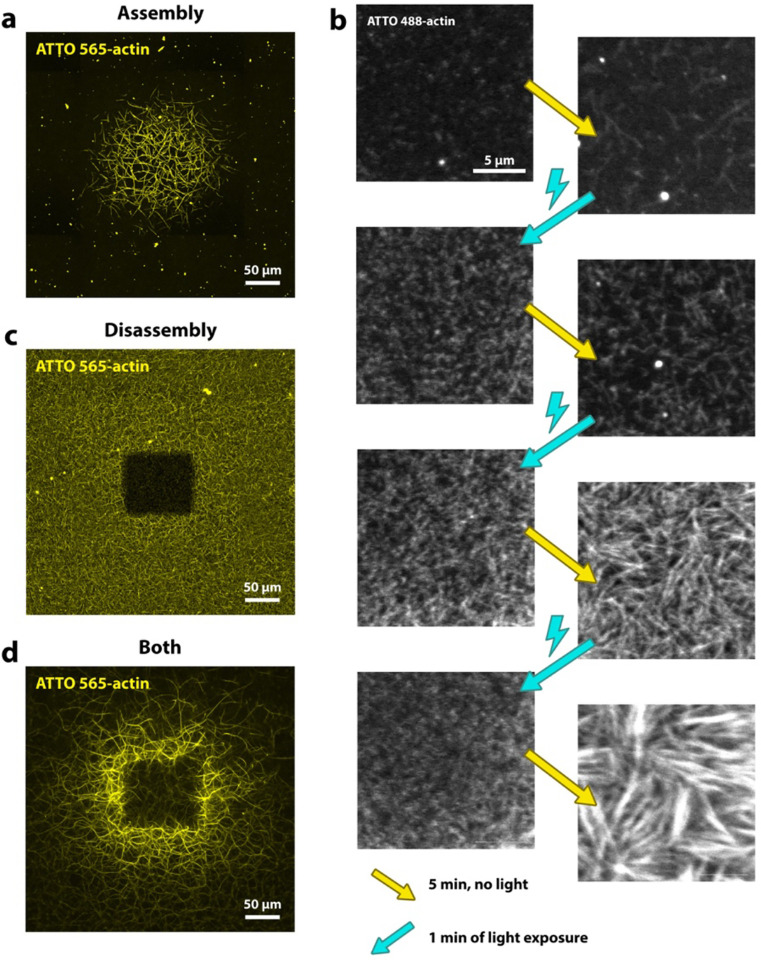
Light controlled actin assembly. **a)** Experiment showing light-induced increase in actin assembly. Condition with low intensity laser exposure and low actin concentration (1 μM). **b)** Intervals of high light exposure followed by rest time with no light exposure. Cyan arrows indicate strong light exposure, which causes filaments to fragment, resulting in numerous visible small speckles. The right column shows images after 5 minutes of no light exposure during which filament elongation occurs, which causes an increase in fluorescence intensity and structures that are visibly more filamentous. **c)** Actin dissembles in the light-exposed region in experiment with higher laser exposure, higher actin concentration (2 μM) and delayed onset of laser exposure. **d)** Combination of increased filament assembly and filament disassembly in experiment with high laser intensity and low actin concentration (1 μM). All images show maximum projections. In all experiments we include macromolecular crowder to induce actin bundling, even though this component is not required (Figure S1).

We use confocal fluorescence microscopy to observe the polymerization of fluorescently labeled actin in vitro. Our experimental setup is simple: we image a standard actin polymerization assay on a confocal microscope, where a square region of the sample, given by the microscope’s field of view, is exposed to light. Interestingly, prolonged exposure to the microscope’s light source causes an increase in filamentous actin (F-actin) in the illuminated region (Figure 1a), which we refer to as “actin printing”. Light-induced increases in F-actin occur within 10–15 minutes of continuous exposure (for more details see Methods section). However, even a single, short, but intense exposure can result in an increase in actin polymerization if the sample is allowed to polymerize for several minutes before and after the light exposure (Figure S2). We can gain some first insights into the underlying mechanism by applying intervals of strong light illumination followed by periods without light: We observe that the light itself does not directly increase actin polymerization, but instead causes the fragmentation of existing filaments into smaller filaments. During the periods between light exposure we then observe an increase in F-actin, which exceeds that in areas not exposed to light (Figure 1b).

## Localized disassembly of actin networks

We can also achieve the opposite effect, in which light disassembles filaments in the exposed region. If we shine light on a sample with an existing network of F-actin, we see a slow disassembly of actin filaments in the immediate light-exposed area (Figure 1c).

Moreover, we also observe this effect in our actin printing experiments under polymerizing conditions, if we increase the light intensity. Above a certain light threshold we note a lack of filaments in the center of the final printed structure (Figure 1d). In these cases we usually observe an initial increase in actin polymerization in the exposed region, followed by network disassembly after several minutes (see Figure S3 and Movie 1). Filament disassembly typically does not extend far beyond the immediate light-exposed area. In contrast, light-induced increases in polymerization spread beyond this area, particularly when F-actin is disassembled in the illuminated area, which then results in the ring-shaped pattern as seen in Figure 1d.

The filament disassembly we observe resembles photo-bleaching, therefore we wanted to rule out that our observations might stem from such photochemical artifacts. Direct evidence comes from the brightfield images of our experiments, which can detect thick actin bundles and confirm the physical lack thereof in the center region of a ring-shaped print (Figure S4).

Time lapse recordings of exposure at high light intensities show a steady fragmentation of filaments into continuously smaller fragments (Movie S2), suggesting that filament severing might also explain actin network disassembly.

## Fluorophore excitation by light result in generation of ROS

As described, with increasing light intensity we see a transition from filament assembly to filament disassembly in the light-exposed region. More precisely, we note a dependency of this behavior on the density of light, both over area and time (Figure S5a–c). Further, actin printing depends on the presence and concentration of fluorophores in our samples (Figure S5d–e). We note that our experiments require light with a wavelength within the absorption spectrum of the fluorophore (Figure S6), indicating that the excitation of fluorophores is critical part of the underlying mechanism.

**Figure 2.**
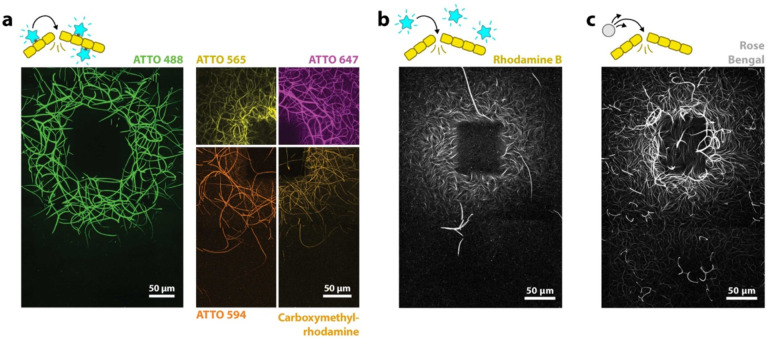
Various types of photosensitive molecules produce similar results. **a)** Ring-shaped prints are achieved with actin with 5 different fluorescent labels. These fluorophores are covalently bound to actin monomers. **b)** Experiment with a fluorophore, which is not attached to actin. **c)** Similar to b), but instead of an efficient fluorophore, we use the photosensitizer rose bengal. Rose bengal produces ROS more efficiently than the other photomolecules and as such was used at much lower concentrations. In b) and c) we still use low amounts of fluorescent labels for actin imaging, however with an excitation spectrum that does not overlap with the excitation wavelength used for printing. All figure panels show maximum projections of the samples.

In most of our experiments, we use fluorophores that are covalently bound to actin monomers, i.e. fluorescently labeled actin, which is commonly used to visualize actin for microscopy. Conveniently, in these experiments, fluorescent actin doubles as a ‘photosensitizer’ for actin printing and as a microscopy label. We see both light-induced assembly and disassembly of F-actin for all different fluorescently labeled actins tested (Figure 2a and Figure S7).

While the use of fluorescently labeled actin simplifies the experiments, we note that fluorophores do not have to be covalently bound to actin molecules: We see both increases in polymerization as well light-induced disassembly when we instead have free fluorophores in solution, which are not bound to actin. Figure 2b shows an experiment with unbound rhodamine B. However, here we have to increase the concentration of fluorophores to achieve results consistent with those with fluorescently labeled actin, indicating that proximity of the fluorophores to the filaments affects the outcome.

Instead of an efficient fluorophore, we can use rose bengal, a compound commonly used as a photosensitizer. While still weakly fluorescent, rose bengal is better known for its ability to produce high amounts of ROS when excited with light. We observe rose bengal to be particularly powerful in our experiments. To achieve light-induced assembly and disassembly, only a fraction of the concentration of rose bengal is necessary compared to fluorophores like Rhodamine (Figure 2c). In experiments in which we do not decrease the rose bengal concentration, we observe an extreme degree of actin disassembly that spreads over an area with a radius of over 1 mm (Figure S8).

Actin polymerization is temperature dependent, therefore we wanted to test whether our actin printing effect is caused by a local increase in temperature. We replace fluorophores with gold nanoparticles, which produce thermal energy when excited within their absorption range.^13^ In these experiments we do not observe actin printing effects, indicating that the effect is not based on increases in temperature (Figure S9).

It is widely understood that fluorophores produce ROS upon excitation with light,^14^ which in turn can oxidize biomolecules such as proteins.^15^ Oxidation has damaging effects, often inducing conformational changes that interfere with protein function. As such, ROS might react with and damage actin monomers within filaments, which could explain our observation of filament severing. To test whether actin printing relies on ROS, we include antioxidants and oxygen scavengers in our samples. We test 3 different reducing agents (β-mercaptoethanol, p-phenylenediamine and FluMaXx, a commercial enzymatic oxygen scavenger system) which either prevent ROS formation or neutralize formed ROS. We find that all three can prohibit actin printing (Figure S10).

## Model based on actin severing

**Figure 3.**
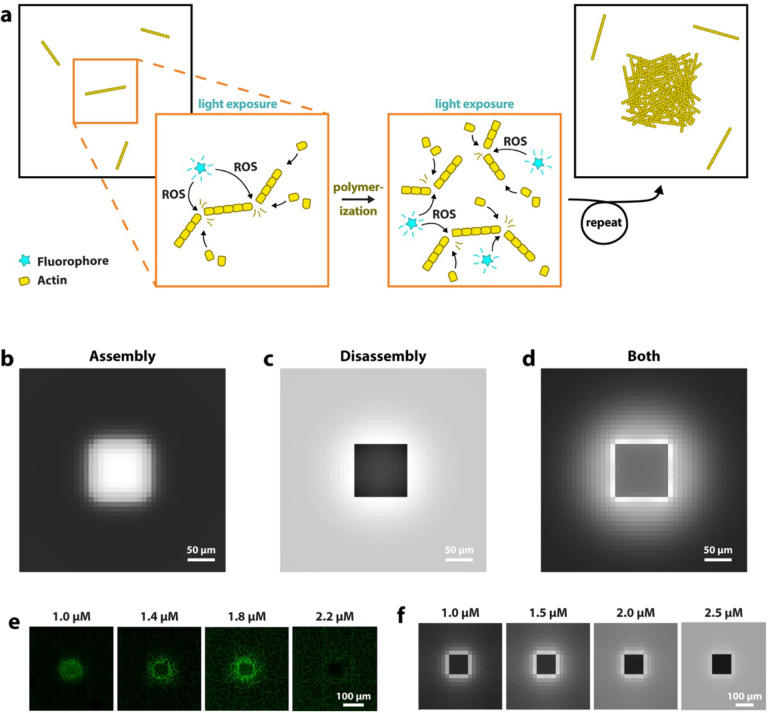
Model based on actin severing. **a)** Schematic illustrating the increase in actin assembly through light-induced severing via reactive oxygen species (ROS). A region within a polymerizing actin sample is exposed to light. Fluorophores produce ROS, which oxidize actin molecules within filaments and thus cause filament fragmentation. Oxidized monomers likely dissociate (not shown) and polymerization continues. Actin assembly is nucleation-limited, thus an increase in the number of filaments increases polymerization. **b)**, **c)** and **d)** show results of our computational model. Simulations correspond to experiments shown in Figure 1 a), c) and d). **e)** Experiments with Atto488-labeled actin at different actin concentrations. **f)** Simulations show a similar concentration-dependent behavior.

Based on our observations, we propose that the fluorophores in our experiments generate ROS upon excitation with light. ROS lead to the severing of actin filaments, most likely due to oxidation of actin monomers within filaments. Given the nucleation-limited nature of actin polymerization, this would explain an increase in actin polymerization: Filament severing results in an overall increase in filaments, and therefore in a greater number of polymerizing ends. Because continued polymerization of existing filaments is kinetically favored over the nucleation of new filaments, an increase in filaments directly correlates with an increase in actin polymerization. The schematic in Figure 3a illustrates this proposed mechanism. Severing also potentially explains network disassembly: If ROS concentrations are high enough, filament disassembly through severing dominates over polymerization and F-actin dissipates in the light-exposed area.

To test the hypothesis that ROS-mediated filament severing underlies both enhanced filament assembly as well as their disassembly, we developed a continuum 2D mathematical model that incorporates actin nucleation and growth. Assuming that local filament severing rate is proportional to local ROS production rate within the illumination region, we evolve the concentration distributions of monomers and filaments, accounting for their length-dependent diffusion. See methods for more details.

In agreement with our experiments, at moderate light intensity, the model shows that fragmentation of actin filaments increases the number of polymerizing filaments and therefore results in an increase in F-actin concentrations in the light-exposed area (Figure 3b and supplementary Figure S11). In further agreement with experiments, light exposure can also lead to an overall decrease in F-actin, because continued fragmentation leads to filaments that are small enough to diffuse out of the illuminated area (Figure 3c). If under polymerizing conditions, once these small filaments reach the periphery, they continue to polymerize and become too long to diffuse back into the exposed region, which explains the ring-shaped pattern of increased F-actin (Figure 3d).

Our simulations give us further insights into whether other, additional factors contribute to light-induced actin network disassembly. Several factors could cause a lack of monomeric actin (G-actin) in the light-exposed area, therefore limiting actin polymerization. In the light-exposed area, G-actin is quickly used up and the diffusion of new G-actin might be rate-limiting. To test this, we speed up simulated G-actin diffusion, but only find this to cause significant differences for very large areas of exposure (Figure S12). Next, we tested whether oxidative damage of actin could reduce the pool of intact G-actin. We note that in our simulations, the pool of oxidized actin stays very small compared to that of intact actin monomers (Figure S13), suggesting that protein damage does not cause a significant decrease in actin polymerization.

We investigate the concentration dependence of actin printing and compare our model to experimental results. Both experiments and simulations show a similar printing behavior across a range of actin concentrations, with less light-induced increases in actin polymerization at higher actin concentrations (Figure 3d). Actin nucleation scales with a power of 3 with increasing actin concentration, as such, at higher concentrations a majority of the actin is already polymerized when light exposure starts. If light exposure initiates at the onset of actin polymerization, actin printing should be more consistent, even at higher concentrations. Experimentally this is not feasible, but we can test this in our simulations (Figure S14).

## Printing complex cytoskeletal networks in 3D

**Figure 4.**
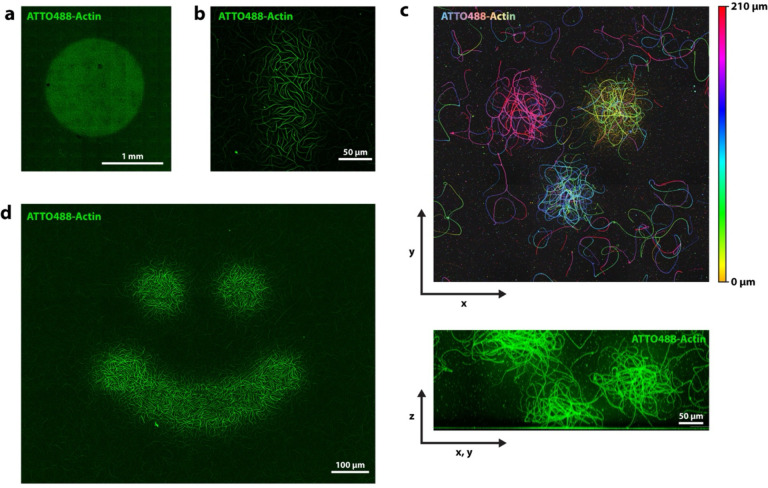
Spatial control over actin assembly. **a)** Light exposure via illumination with a widefield microscope. A large circular area of ~1.6 mm in diameter was exposed, leading to an additive print of roughly the same size. **b)** Light exposure via confocal laser scanning microscope of a small region of 60 μm × 140 μm in size. **c)** Confocal microscopes allow for control of actin polymerization in 3D. Shown are three actin patches polymerized by focusing the confocal beam to 3 different z-heights. Top image: false color image, color represents height in z. Bottom image: side view (projected diagonally). **d)** Arbitrary patterns such as this smiley shape can be printed.

We find the printing effect to be scalable and to allow for patterns in various shapes and sizes. In Figure 4a and b we show printed structures between ~ 1.6mm and ~ 60 μm in width. Figure 4a and Figure S15 show large actin prints achieved with a widefield microscope, unlike all other shown results which were conducted on laser scanning confocal microscopes.

Our technique allows for control over actin assembly in 3D: Using the confocal microscope, we can expose the sample at different positions in z, which results in patches of actin being printed at different z-heights. Figure 4c shows a false color image indicating the position in z, of an experiment where we exposed the sample in 3 independent locations at 3 different z-heights.

Rather than limiting illumination to simple squares or circles, we can project more complex patterns onto the sample. As an example, Figure 4d and supplementary Figure S16 show the sample illuminated in a “smiley” pattern.

**Figure 5.**
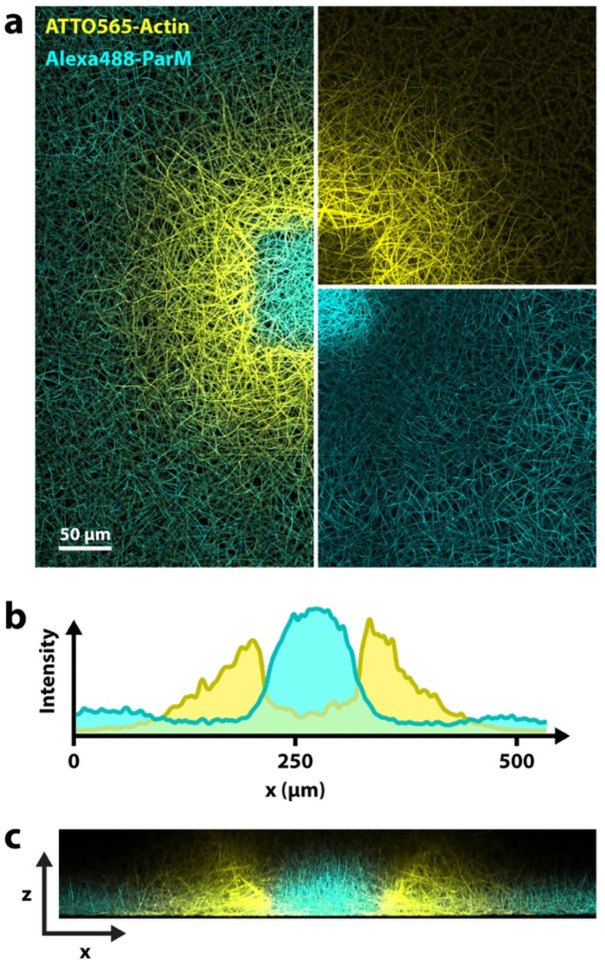
Multi-color printing. Experiment with two types of actin: Eukaryotic actin and the bacterial actin homolog ParM. (Maximum projection.) Both are affected by laser excitation and can be printed. In this case, ParM polymerization was enhances in the center (additive print) and actin polymerization in the periphery (ring-shaped print). **a)** x-y-view with both channels combined on the left and split channels on the right (top: actin, bottom: ParM). **b)** Fluorescence intensity profile measured horizontally across the center. **c)** Side view (x,z) cross section of the sample. (Maximum projection of the light-exposed region.) Different morphologies of the two different cytoskeletal

We find that not only eukaryotic actin is affected by ROS-induced severing, but also the bacterial actin homolog ParM. By combining eukaryotic actin and ParM, we can achieve what we call multi-color printing. We combine ATTO 565-labeled actin and Alexa Fluor 488-labeled ParM and exposed the sample with both excitation lasers at high intensities, which results in an alternating pattern, with ParM in the exposed area, surrounded by an actin ring-print, followed by ParM further away (Figure 5). As Figure 5b confirms, we observe a type of anti-correlation, where the concentrations of one type of polymer are low in regions dominated by the other (also see Figure S17). While we show one specific case here, different variations of this experiment are possible. In supplementary Figures S18 we highlight more observations from these experiments with both biopolymers. Movie 1 and supplementary Figure S19 show the time course within the exposed region of such an experiment, which also unveils some interesting aspects about these experiments.

## Discussion

In this work we show that via the excitation of fluorophores we can control the assembly and disassembly of the cytoskeletal protein actin in three dimensions, including the capability for ‘multi-color’ printing. Our experimental data and simulations support a mechanism in which ROS are produced by these photosensitive molecules, which then fragment actin filaments. We think our findings are significant for various fields. The ability to 3D print actin hydrogels introduces new possibilities for biomaterials science, while the underlying mechanism of growth via fragmentation could be applicable to other materials. Further, our results are a consideration for experiments involving fluorescently labeled actin, where this phenomenon could lead to artifactual effects. Moreover, the controlled manipulation of actin network geometry could serve as a valuable tool for in vitro reconstitution studies, similar to existing surface patterning techniques.^16–18^ Perhaps most importantly, we believe this ROS-dependent mechanism likely affects actin remodeling in living cells, where all requirements are met for it to take place.

In previous literature, both in vitro and in vivo, we find reports that support our findings or observations that can be explained through our model. In vitro, fluorescently labeled actin exposed to light has been observed to cause an increase in actin polymerization, which previously was either attributed to the light^19^ or the fluorescent label alone^20,21^ but not the combination thereof. In vivo, correlations between ROS and actin polymerization are well known and the importance of redox regulation of actin has become of scientific interest.^7–9,22,23^

Several studies show that depleting cells of ROS leads to actin cytoskeleton disassembly and impedes actin-dependent functions such as cell migration,^24,25^ wound healing^26^ and neuronal growth^27,28^. Supplementation with external ROS can rescue this actin and cell function loss.^29^ Most similar to our experiments in vitro, actin tagged with an engineered ROS-generator causes a drastic increase in F-actin in various types of cells.^30,31^ Many older studies on oxidative stress externally add ROS (typically H_2_O_2_) to cells like macrophages^32–35^, astrocytes,^36^ and drosophila cells^37^ and describe increases in F-actin by up to 8.5 fold^37^. Further, there is a strong correlation between mitochondrial dysfunction, ROS production, and actin network density: Damaged mitochondria produce high levels of ROS, leading to ‘acute damage-induced actin’^38,39^ and certain environmental changes have been shown to correlate with both increases in mitochondrial ROS and peripheral actin^40^.

The above studies link increases in ROS to increases in actin, yet none offer conclusive explanations for this connection. There are numerous reports of a negative correlation between ROS and actin, which typically are credited to the general destructive nature of ROS. Excitingly, both actin assembly and disassembly can be explained by our model via ROS-induced severing. We assume fragmentation can both enhance polymerization as well as depolymerization, depending on which process is favored by the current environment. A second effect is that fragmentation increases filament diffusion, which explains how, even in our experiments under polymerizing conditions, local disassembly can be induced. In cells, the consequences of ROS-induced fragmentation are likely dependent on many different factors, including the availability of actin monomers and specific actin regulators. A relevant comparison is the actin-severing protein cofilin, which has indeed been shown to increase actin polymerization^41–43^ but usually collaborates with other proteins to promote filament disassembly.^44^ Thus, we speculate that ROS-induced severing might have extensive but complex implications for actin remodeling in cells. And indeed, many recent studies describe the effects of ROS on actin rather broadly, i.e. that it affects actin remodeling, reorganization or increases actin dynamics across various cellular processes,^9,45^ such as cell migration,^46–48^ cell adhesion^49–53^ and cell growth^54^.

Much of actin research has focused on the numerous actin accessory proteins that specifically bind and regulate actin polymerization and network architecture, offering an explanation for the remarkable versatility of the actin cytoskeleton. Our findings broaden the understanding of actin organization beyond specific protein interactions, suggesting that ROS, which are ubiquitous in cells and integral to cell homeostasis, add another layer of regulation to actin dynamics and remodeling.

## Supplementary Material

Supplement 1
