# Supplementary material for: 3D printing cytoskeletal networks: ROS-induced filament severing leads to surge in actin polymerization
Source: bioRxiv. 2025 Mar 20:2025.03.19.644260. Preprint. [Version 1] doi: 10.1101/2025.03.19.644260 (PMC11957145; doi:10.1101/2025.03.19.644260)
Supplement: Supplementary file 1 [file media-1.pdf]

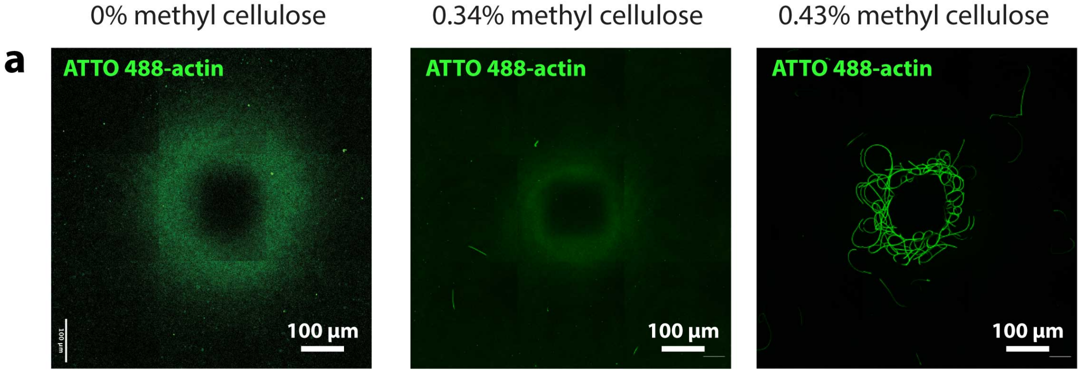

Figure S1: Actin printing in dependence on crowder concentration. **a)** Actin printing can be observed in experiments without any crowder (left panel): we still see a donut-shaped pattern even without methyl cellulose in experiments with high laser power, i.e. an increase in F-actin surrounding the exposed region. However crowder (here in the form of methyl cellulose) affects the results. With increasing methyl cellulose, the printing becomes less diffuse, possibly due to a higher viscosity and reduced diffusion. If we increase crowder concentration even further, we cross a bundling transition and actin bundles start to appear.

a

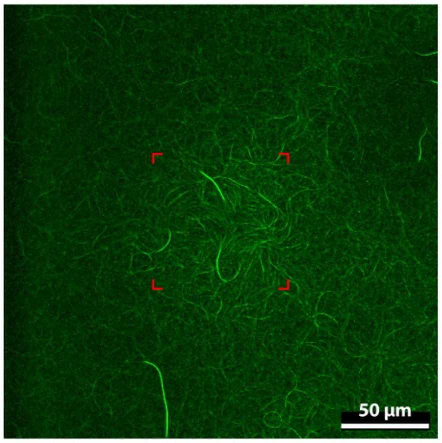

Figure S2: a) Light exposure leads to increase in actin polymerization even after halt of exposure. Even a very short exposure of 10 seconds leads to an increase in actin. Shown is a sample with 1.5 µM actin and 0.01 µM rose bengal. The samples was left to polymerize for 15 minutes, then the region in the center (red mark) exposed at 514 nm light for 10 seconds and left to polymerize for another 10 minutes. Even with such a short exposure time, a clear increase in actin can be seen in the exposed region.

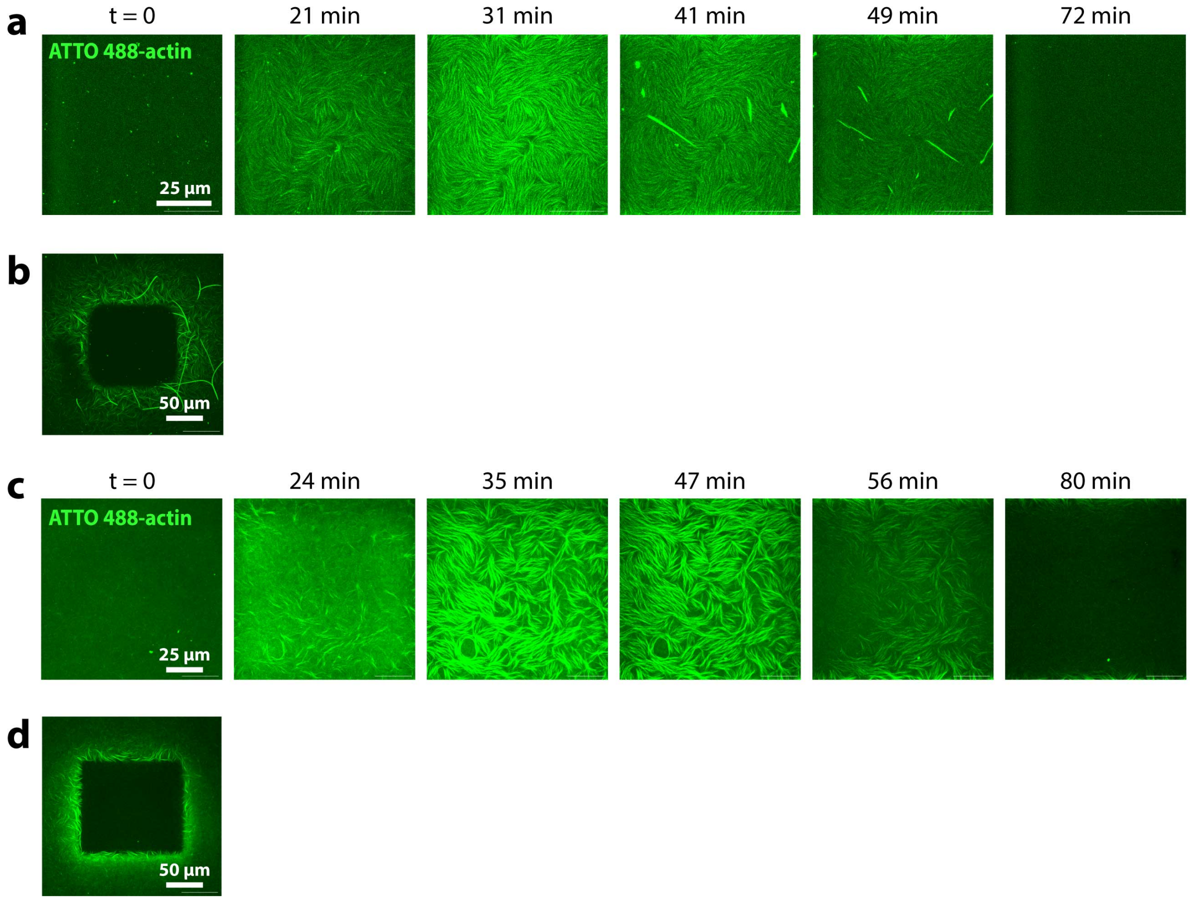

Figure S3: Development over time of actin prints exposed for a prolonged time. a) and c) show the development over time of the directly light-exposed area. Shown are the images acquired via the light exposure: Most of our printing is simply done by recording a time lapse where the excitation light for acquiring fluorescence images doubles as the exposure light for printing. As such, shown are frames from the acquisition that cause the printing. b) and d) show a zoomed out view of the area after the respective light exposures shown in a) and c). The two cases [a) and b) vs c) and d)] were performed under identical conditions with the only difference being the size of the exposed area (i.e. the zoom setting on the laser scanning confocal microscope). In the first case, a smaller are was exposed, as indicative by the scale bar. Because the laser power output was identical in both cases, the laser intensity per area is higher in the first case, leading to a faster progression of actin assembly and disassembly compared to the second case. We see this progression from actin bundles appearing (additive print) to them disappearing (subtractive print) (which results in a donut shaped morphology) only up to a certain laser intensity. Our simulations suggest that in the case of donut-shaped prints, there is always an intermediate step in which the actin concentration in the center is higher than in adjacent regions. In our experiments, at higher laser intensities, the donut morphology seems to immediately emerge, without the intermediate step of an additive print. At first, with increasing laser intensity, the intermediate increase in actin becomes less noticeable, because no bundles form in the exposed region. Often we see an increase in

fluorescence intensity (increase in F-actin), but no bundling. The lack of bundling is likely due to the fact that filament bundling both depends on filament concentration and filament length. This means that filament concentration can be increased, but due to fragmentation, filament lengths are too short for bundling to happen. Figure S24 shows this effect clearly. At even higher laser intensities, no increase in actin is observed at all as an intermediate step (See Figure S13b). We think this is likely due to photobleaching.

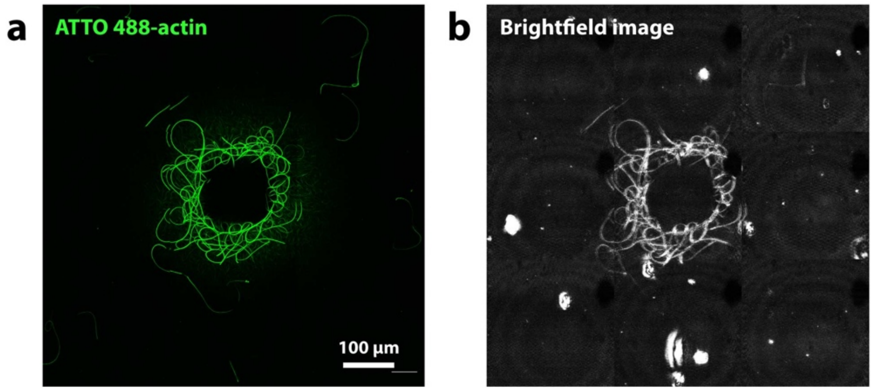

Figure S4: a) Fluorescence microscopy image and **b)** brightfield image of a ring-shaped print. The brightfield image is heavily processed, which allows us to see thick actin bundles. The similarity of the two images indicates that photo-related artifacts like photobleaching are not a major contributor to either additive or subtractive printing.

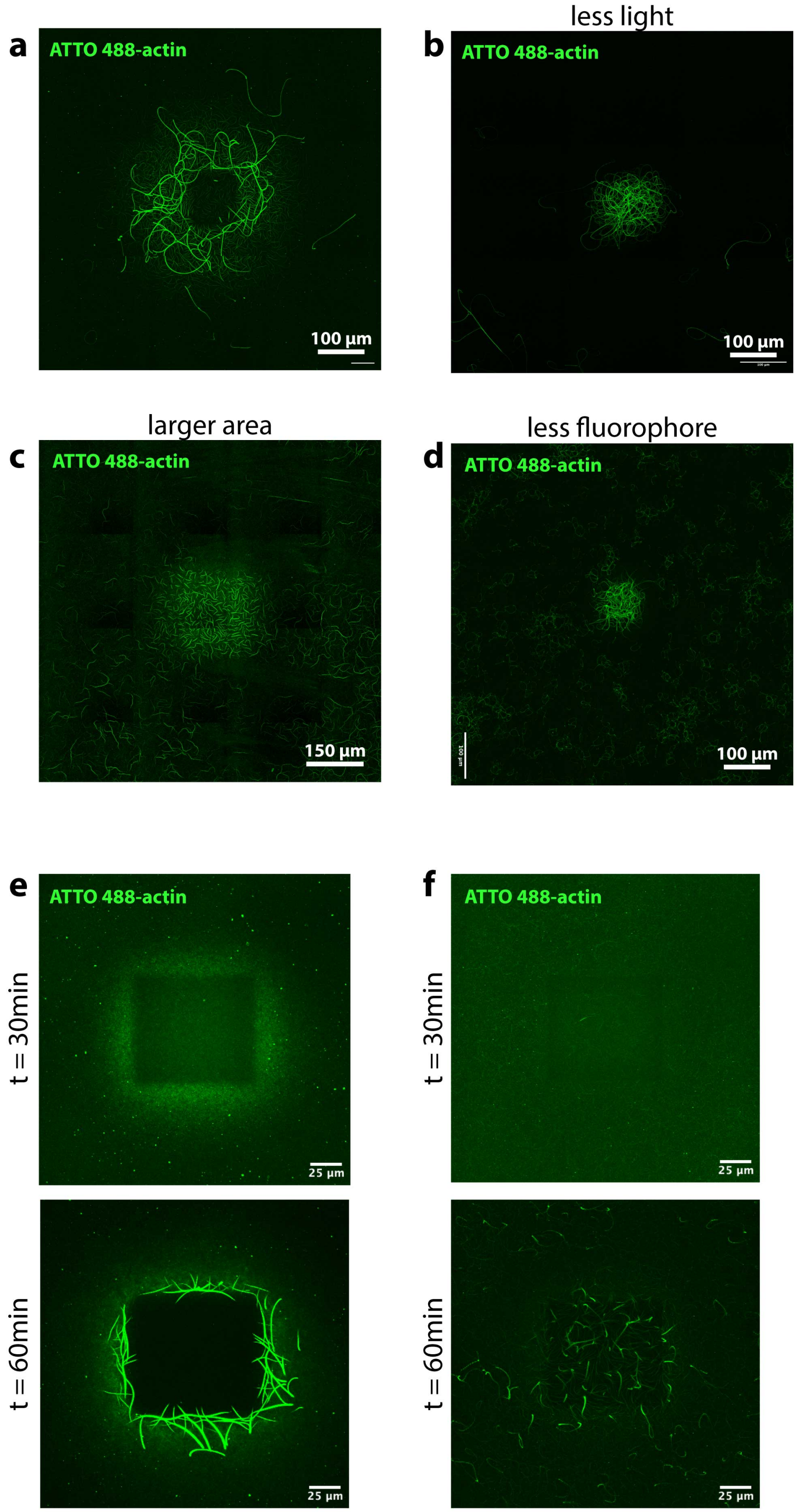

Figure S5: With decreasing ROS generation we see a transition from a ring-shaped print pattern with actin disassembly in the center, to a purely additive print. **a)** "Control"

experiment, similar to donut print experiments in other figures (Fig. 1d, Fig 2a-c, Fig 3a). 1.5 µM actin of which ~70% are fluorescently labeled (ATTO 488-actin), continuously laser exposed, exposed area is ~100 µm x 100 µm in size. **b)-c)** Experiments in which ROS generation (over space or time) is decreased, result in additive print. Each image shows experiment in which one parameter was adjusted compared to a). **b)** Experiment with less light exposure. Same laser intensity, but only 25% of the time the laser is on; 75% off-time. **c)** A larger area is light exposed, which, using a confocal microscope, results in a lower light density. Exposed area is ~270 µm x 270 µm. **d)** Experiment with less fluorescently labeled actin (~23% ATTO 488-actin, ~77% unlabeled actin). **e)** and **f)** Direct comparison between experiment with ~70% labeled actin (e)) and ~23% labeled actin (f)) after two different times of exposure.

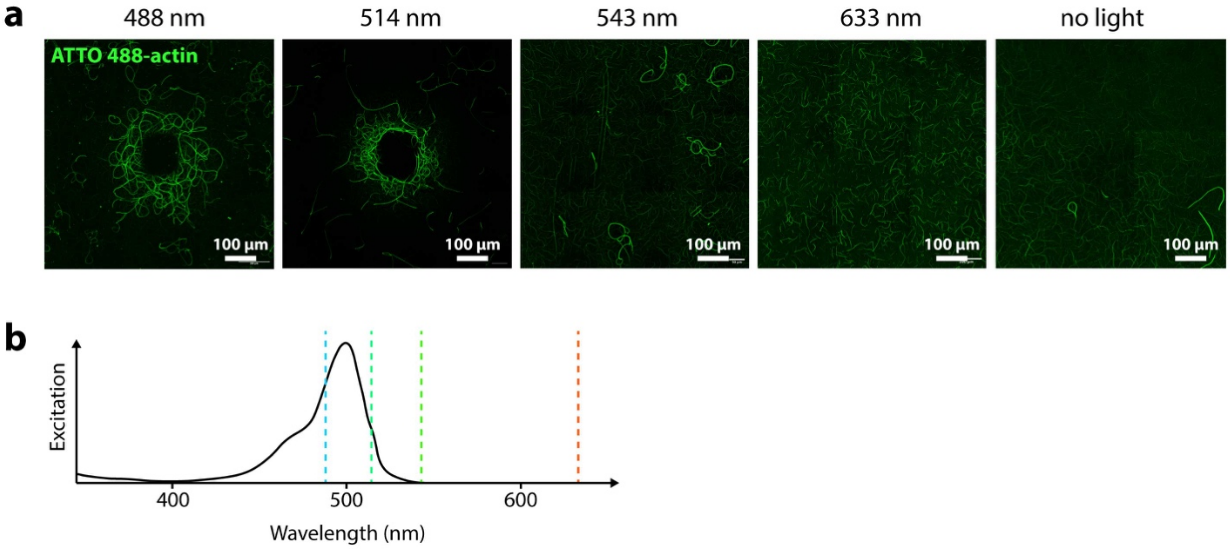

Figure S6: a) Actin printing experiments with high laser power and 1.5 µM ATTO 488-actin, similar to Figure 2a. Compared are experimental runs in which sample is exposed to lasers of different wavelengths. Printing is only observed when wavelengths are used that are within the excitation spectrum of the fluorophore (see b)). **b)** Excitation spectrum of ATTO 488. Adapted from (*AAT Bioquest, Inc. (2024, November 11). Quest Graph™ Spectrum [Atto 488]. AAT Bioquest. https://www.aatbio.com/fluorescence-excitation-emission-spectrum-graph-viewer/atto_488*). Four excitation wavelengths from a) are marked with dotted lines. Normalized absorption for 488 nm is 69%, for 514 nm is 39 % and for 543 nm and 633 nm are both <1%.

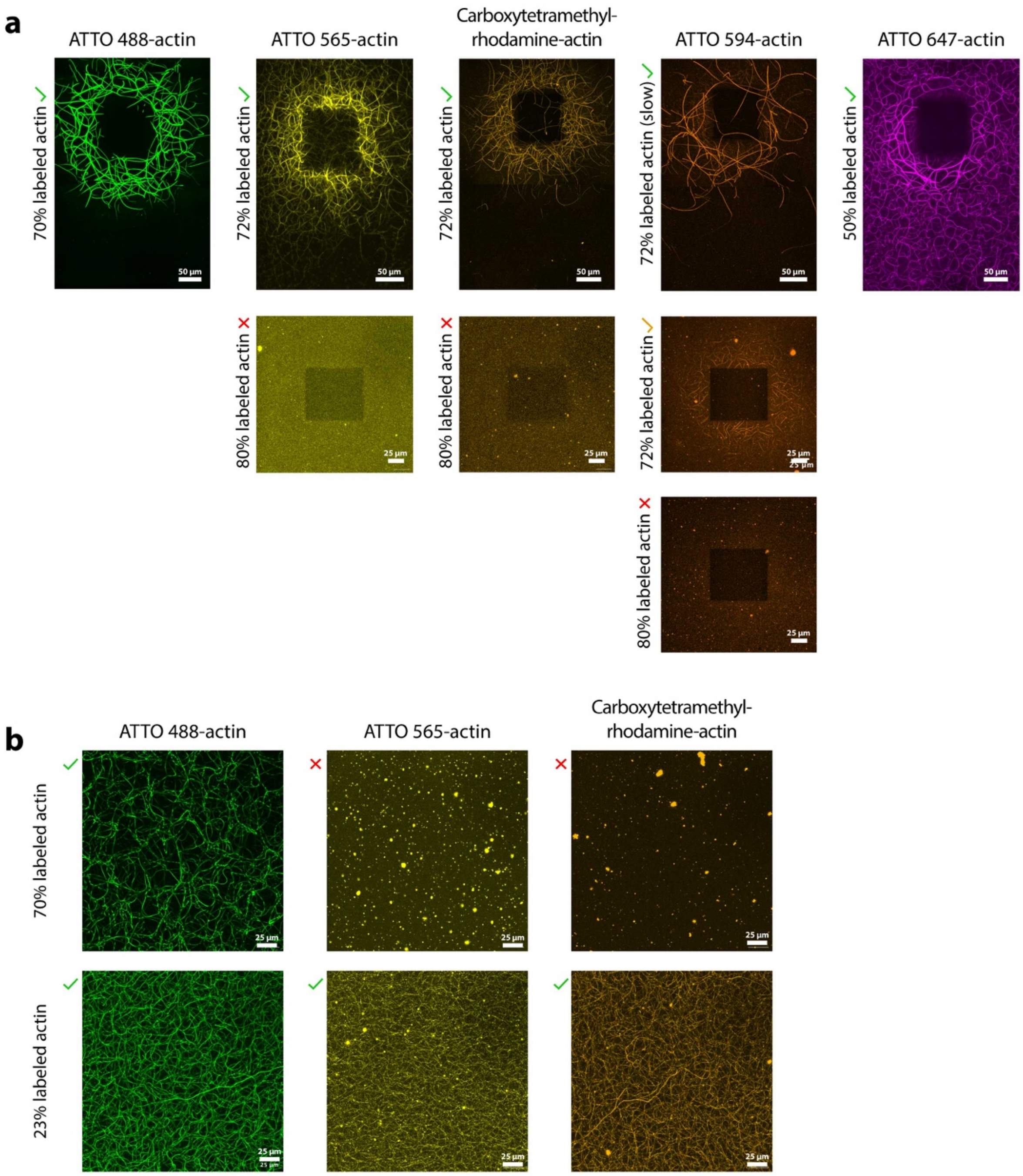

Figure S7: a) Different fluorescently labeled actins at various degrees of labeling. We observe differences between fluorescent labels and note that the ratio of labeled to unlabeled actin needs to be chosen carefully to achieve consistent outcomes. The first image with 70% ATTO 488-actin shows a sample in which 80% of the actin is ATTO 488-actin and 30% is unlabeled actin. The highest degree of labeling (most bottom image) for each label shows an experiment with the commercial product in its stock degree of labeling, i.e. not diluted with unlabeled actin. This degree of labeling varies depending on the type of fluorescently labeled

actin. Only for ATTO 488-actin and ATTO 647-actin we were able to use these highest degrees of labeling successfully. **b)** Not all fluorescently labeled actins can be used at high ratios of labeled to unlabeled protein. ATTO488-actin can be used at high fractions, while ATTO 565-actin and Carboxytetramethylrhodamine-actin fail to polymerize at these high fractions.

a

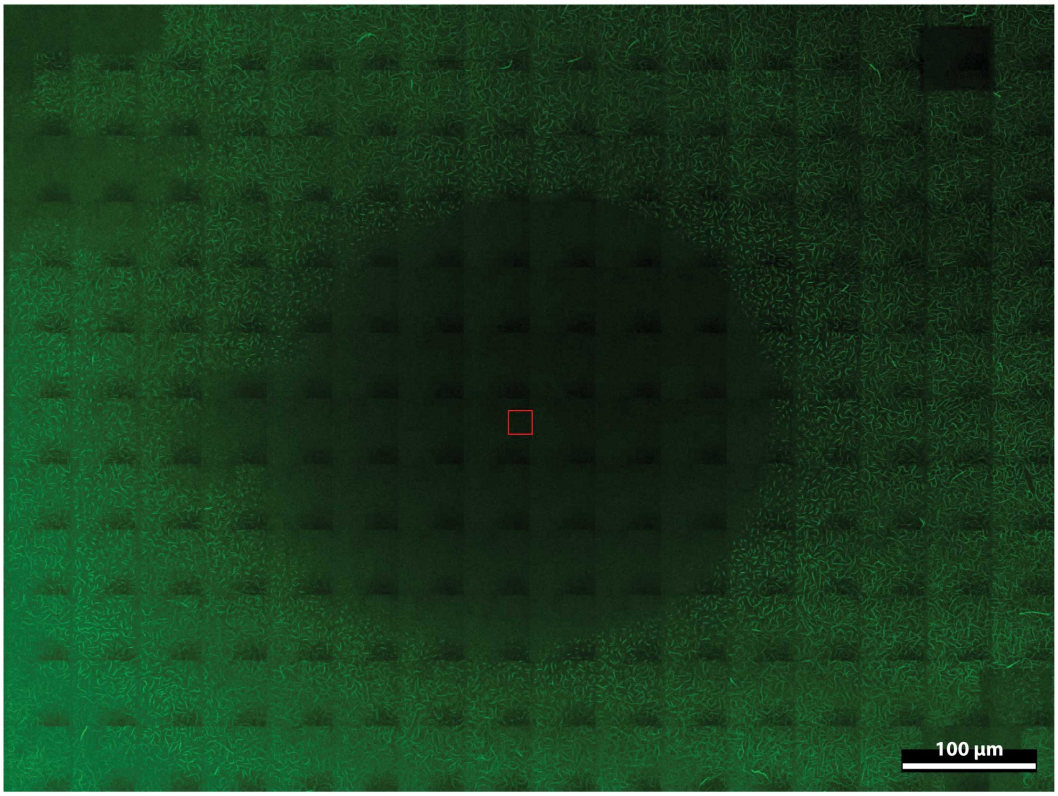

Figure S8: Subtractive printing can be diffuse. a) Experiment with rose bengal, with rose bengal concentrations similar to concentrations of fluorophores in our other experiments (1.5 µM). Due to rose bengals propensity to produce ROS very efficiently, ROS is produced in large concentrations in this experiment. This high concentration of ROS is allowed to diffuse further, hence fragmenting actin far away from the light-exposed area.

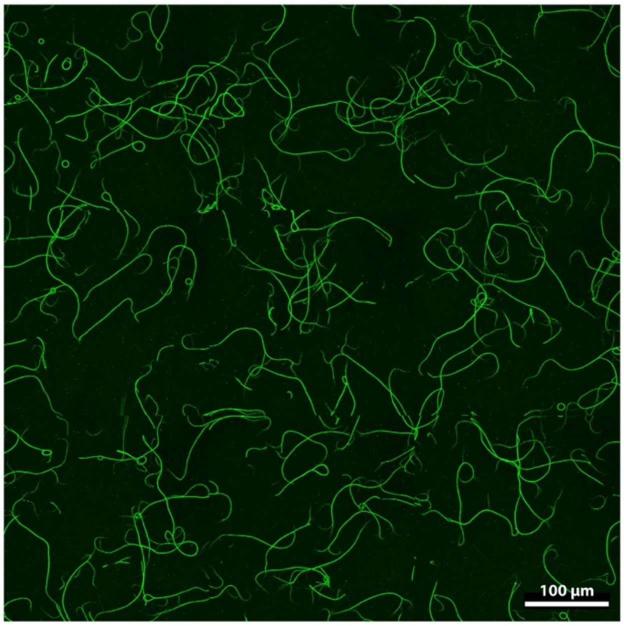

Figure S9: Experiment with gold nanoparticles. Gold nanoparticles produce thermal energy when excited within their size-specific excitation wavelength spectrum. We performed this experiment to test whether actin printing might be caused by increases in temperature, as gold nanoparticles should be more efficient at increasing temperature compared to fluorophores. We do not see any effect on actin polymerization or disassembly when we excite gold nanoparticles via laser excitation. Experiment with 1.5 µM actin (35% ATTO 488-actin), 0.43% methyl cellulose, 2 mM ATP-γ-s. This sample has with $4.7*10^{12}$ particles/mL of 5 nm gold nanoparticles, which have excitation maximum between 510 nm and 520 nm. Sample was exposed at 514 nm for 150 min.

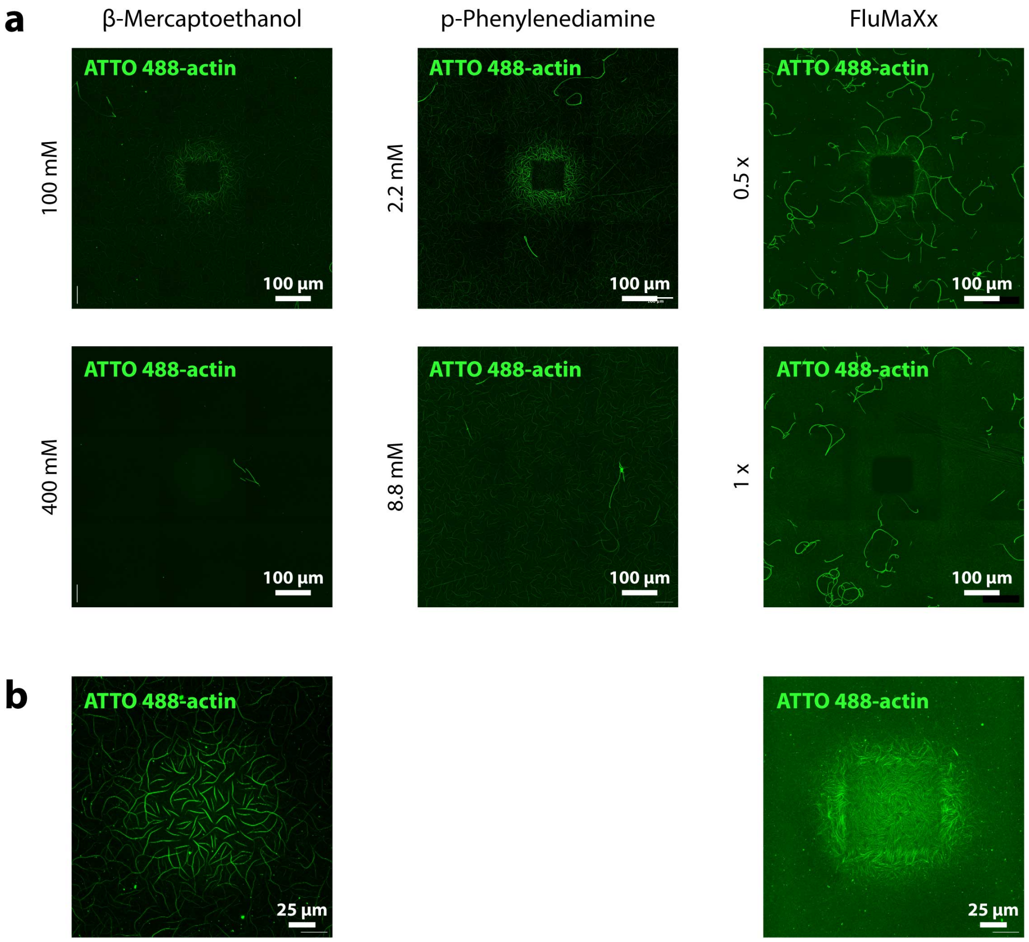

Figure S10: Experiments with antioxidants and oxygen scavenger. Conditions with 1.5 µM ATTO488-actin and high laser intensity exposure, similar to experiments in Figure 1d, 2a-c and 3a, however here with the addition of antioxidants/oxygen scavenger and with a longer exposure time. β-Mercaptoethanol (BME) and p-Phenylenediamine (PPD) are antioxidants, while FluMaXx is a commercial oxygen scavenger system. FluMaXx consists of the enzymes glucose oxidase and catalase and glucose as a substrate. Results with BME are also shown in Figure 3c. In each experiment the sample was exposed for 2.5 hours, which is a particularly long time. Here we wanted to test for both additive printing and subtractive printing, hence we chose such a long exposure time. b) If exposed for a shorter time, positive printing can be observed, similar to other cases with lower ROS-production rate (see Figure S9). Shown here is an experiment with 100 mM BME (left) and 0.5 x FluMaXx (right) after 45 min and 30 min of exposure respectively. In both cases additive printing is prevalent. Without these compounds (BME and FluMaXx) these exposure times would result in a donut-shaped print (subtractive in the center). The exposed regions are the same size as in a), but note the smaller field of view in both images compared to a).

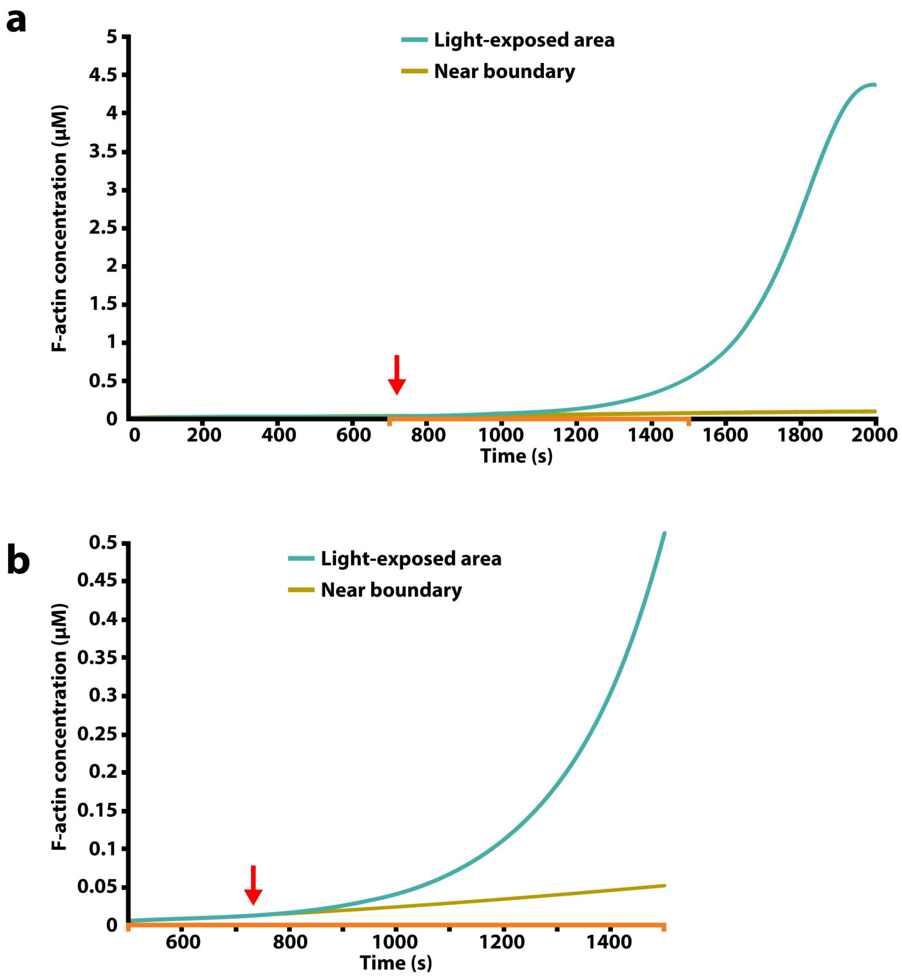

Figure S11: F-actin concentration over time in simulation of light-increased actin polymerization. a) Model with low light intensity, exposing an area of 75 x 75 μm in a sample of 225 x 225 μm. Initial G-actin concentration is 0.75 μM. Light exposure starts at t= 720 s (red arrow). Cyan curve shows the F-actin concentration in the very center of the light-exposed region. Yellow curve shows F-actin concentration near the boundary of our simulated sample, which is mostly unaffected by the light-exposure. **b)** Zoom in on time interval between t = 700s and t = 1500s of a). Initial exponential increase in F-actin can be seen, after light-exposure and before G-actin depletion slows down polymerization.

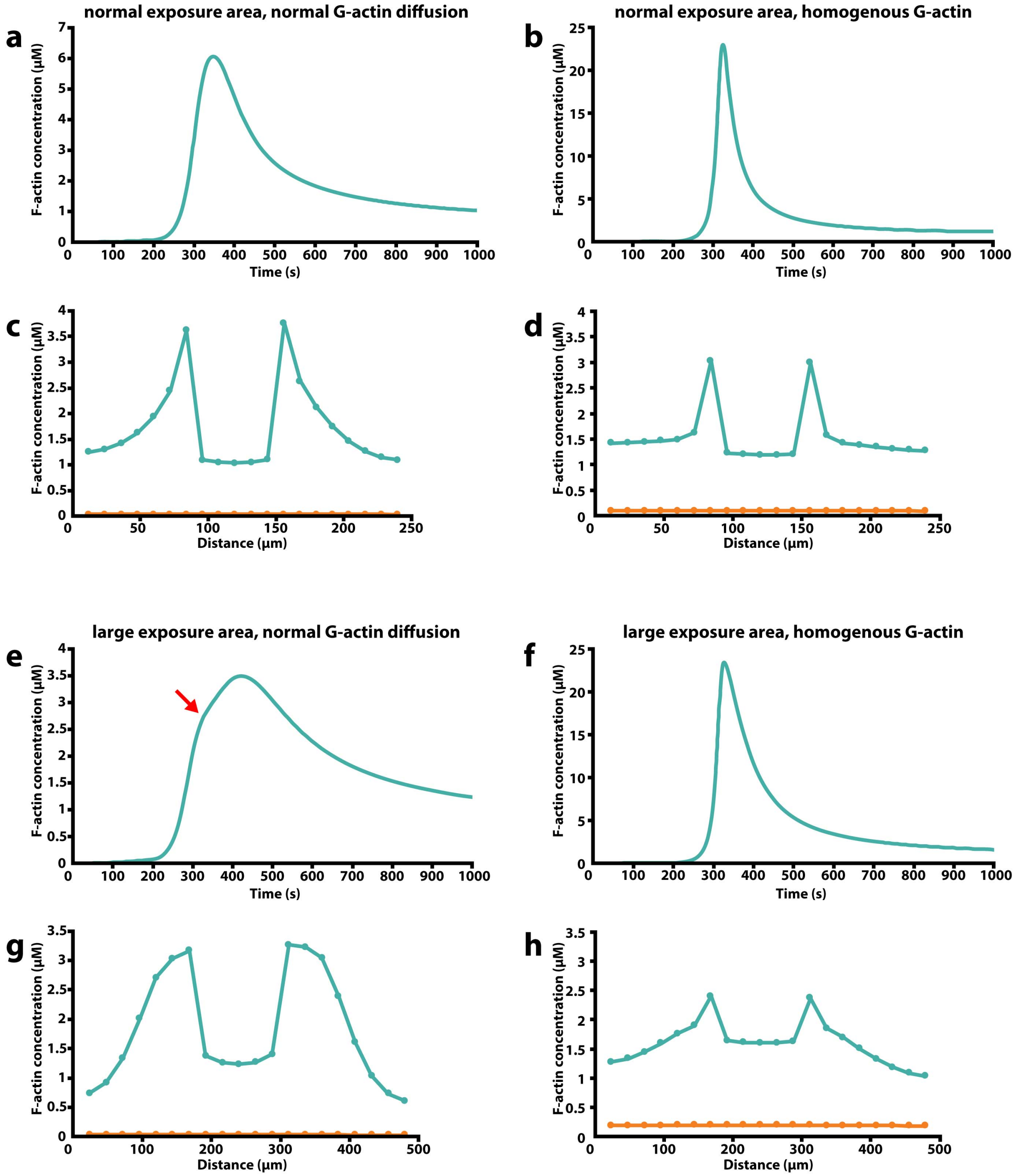

Figure S12: Effect of G-actin diffusion on printing. We design a simulated experiment so that G-actin concentration is homogenous across space at all times, essentially simulating a diffusion coefficient that is infinitely high. **a), b), e), f)** show F-actin concentrations over time in the light-exposed region. **c), d), g), h)** show the concentration profile across the sample at t = 1000s for all conditions. The directly light exposed region is visible as a dip in F-actin concentration in each plot. Four simulations are shown. The first column shows two experiments with normal G-actin diffusion. The second column shows two experiments in

which we homogenized G-actin concentration across space. The top two simulations show a smaller simulated sample with a smaller light-exposed area. The bottom two simulations show a larger sample with a larger light-exposed area. As expected, the increased G-actin diffusion accelerates actin polymerization, as the pool of G-actin is constantly replenished. Note the vastly different y-axis a scales in figures a), b), e) and f). For smaller exposed areas, the concentration profile at 1000s changes relatively little when comparing the two different G-actin diffusion scenarios [c) and d)]. For larger exposed areas, G-actin diffusion affects the outcome more strongly [g) and h)]. Also note a kink that can be seen in the F-actin concentration curve (red arrow) in g), which shows the complete depletion of available G-actin, which disappears for high G-actin diffusion [f)].

Figure S13:

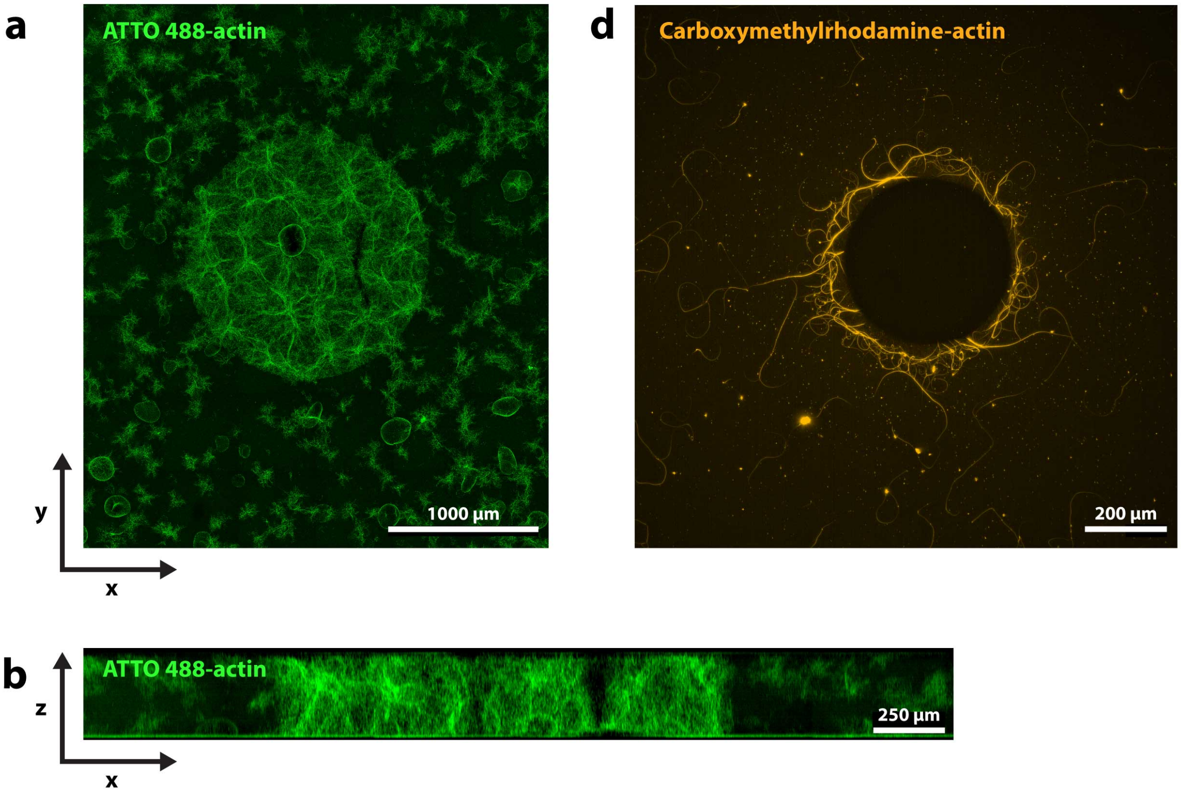

Figure S15: Large actin prints. **a)** Actin print similar to Figure 4a, but with a lower actin concentration (1.2 µM, 23% ATTO 488-actin) and exposed for a longer duration at low light intensity. **b)** Side view of the sample (maximum projection), shows that the print expands in z, resulting in a large printed volume. **d)** Actin print performed on a widefield microscope with carboxymethylrhodamine-actin. Sample was exposed for 5 hours using 475 nm and 555 nm light simultaneously. Sample with 1.7 µM actin (50% carboxymethylrhodamine-actin).

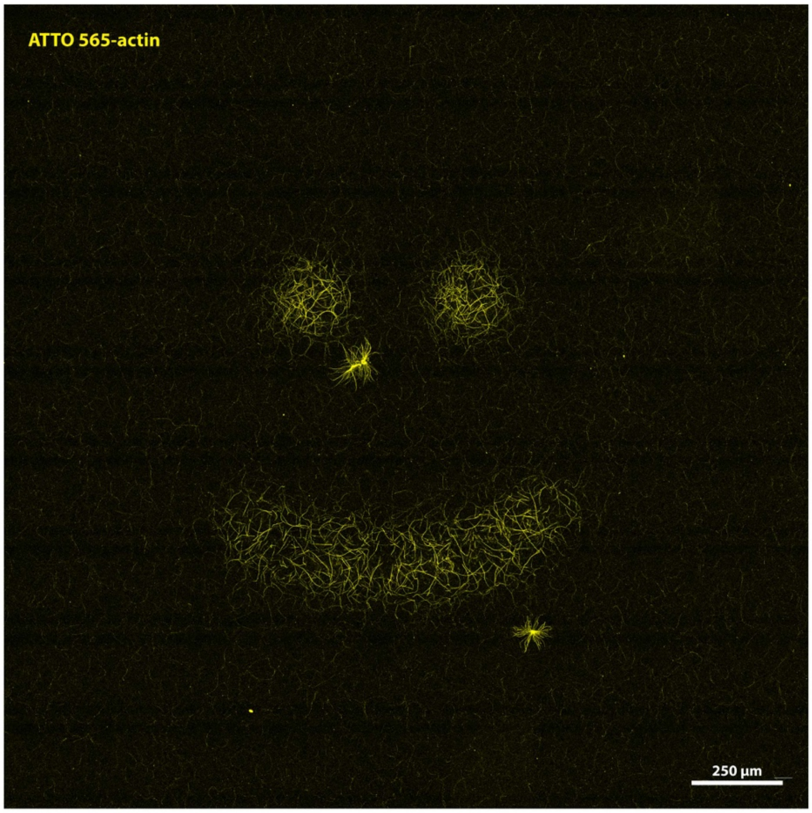

Figure S16: Additional example of smiley-shaped actin print, similar to Figure 4d, but using ATTO 565-actin.

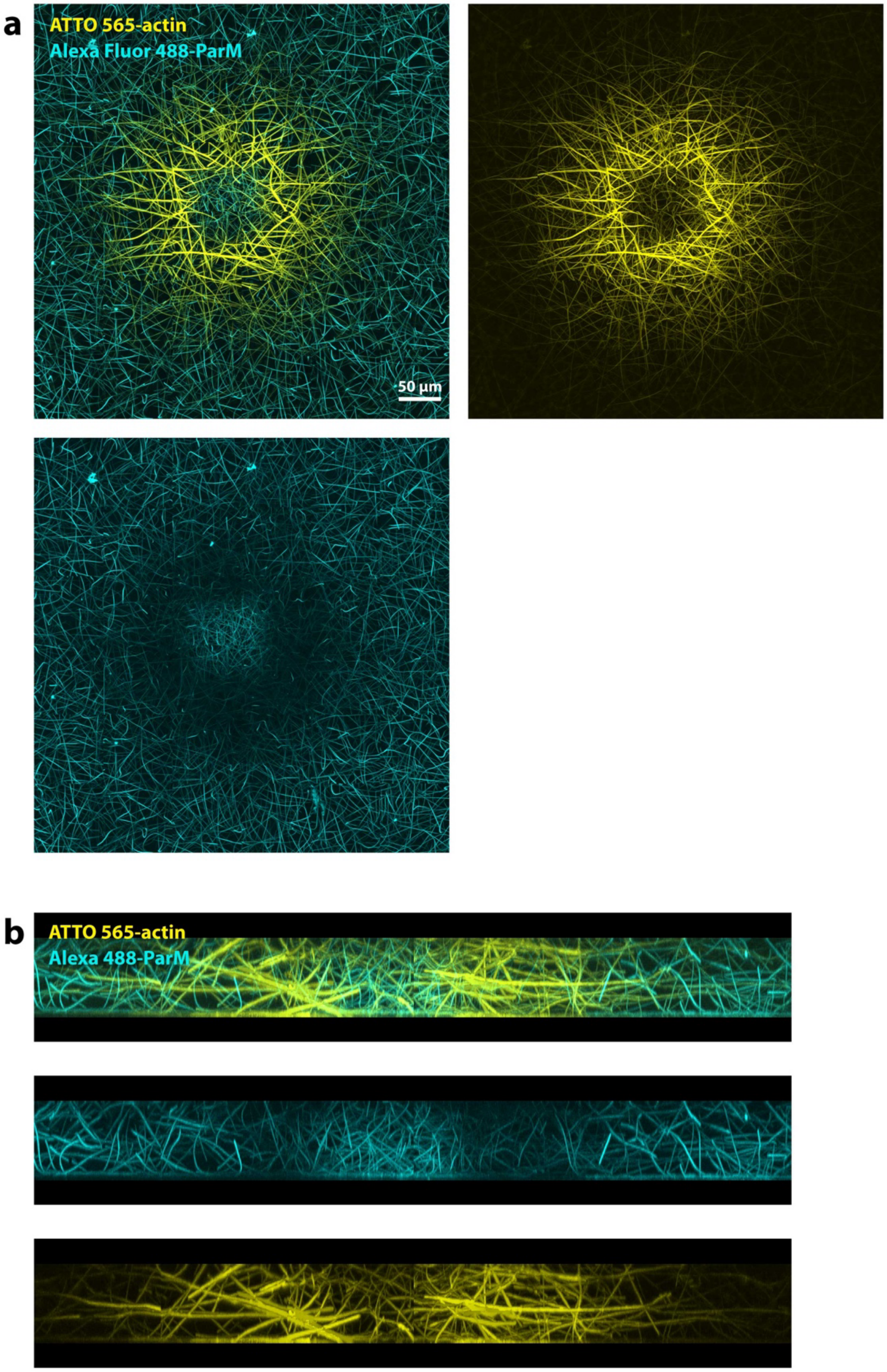

Figure S17: Additional example of ParM-actin multi-color printing. Experiment similar to Figure 5, but slightly different experimental parameters. (More crowder, smaller exposed area, less light intensity, prolonged exposure.) **a)** Similar to the experiment in Figure 5, we see a decrease of ParM in the region with actin. One possible explanation for this decrease could be

fluorophore quenching, if ATTO 565 and Alexa 488 are in close proximity. As we show in Figure S18a the fluorophores can be colocalized within bundle. The example shown here is the same as shown in Figure S18d, where we see very little colocalization, indication that the decrease in ParM signal is not due to fluorophore quenching. **b)** Side view cross-section of the sample. Shown is a maximum projection of the center region (80 µm thick) of the sample. Interesting here are the quite different morphologies of ParM and actin networks. Actin seems to form thicker bundles, which are mostly horizontally (x-y) oriented, while ParM bundles are thinner and more vertically (z) oriented. The actin print shown in Figure 5c shows a similar effect, however not as pronounced. We think this demonstrates an intriguing aspect in respect to possible biomaterial applications, as this demonstrate that our “multi-color printing” can not only be used to print different fluorescent labels, but also biofilaments with different physical properties and possibly different functionalities.

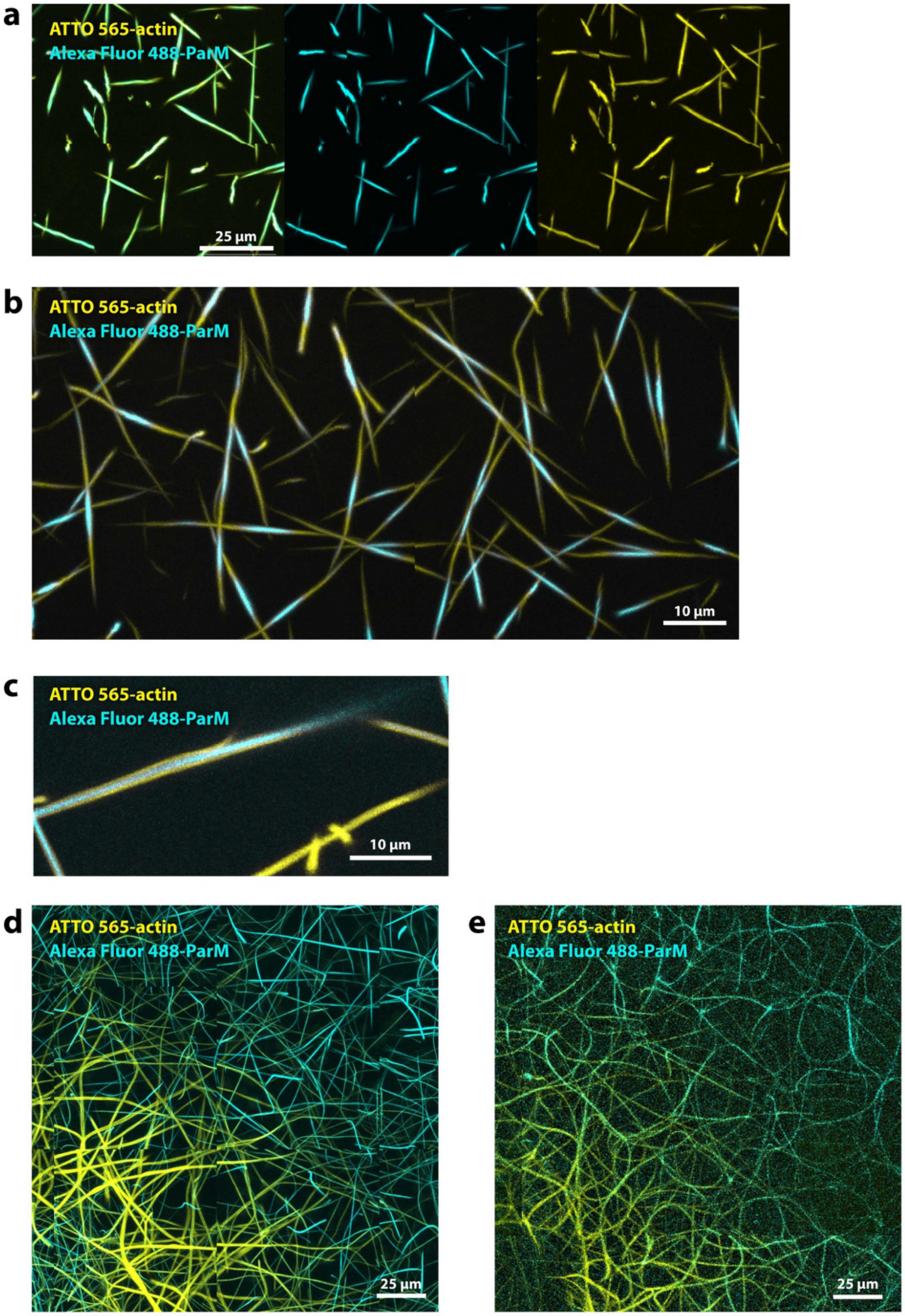

Figure S18: Colocalization of actin and ParM. **a)** We often see colocalization of actin and ParM within one bundle. We assume that actin and ParM polymerize independently, i.e. form separate filaments, but that bundling does not discriminate between the two types of filaments, hence within bundles both types of filaments coexist. **b)**, **c)** Due to the different

polymerization kinetics of the two filaments, we sometimes see bundles within which the two types of filaments appear separated and form patterns. This can happen longitudinally (b)) and laterally (c)). In both of these cases, the initial bundle formed as a bundle of ParM filaments (cyan), and then grew through attachment of actin filaments (yellow). In b) bundles form with a core rich in ParM and two ends rich in actin, however in some cases several of these actin-ParM-actin bundles coalesce later on and thus bundles form with a repetitive striped pattern. **d)**, **e)** ParM and actin printing experiments (similar to experiment in Figure 5) in with the transitions between actin-rich and ParM-rich regions differ. Panel d) shows a case in which we see very little colocalization of ParM and actin within bundles. ParM and actin bundles are physically separated, with few exceptions. Panel e) shows an experiment in which the large-scale printed pattern looks very similar, but the transition from actin to ParM can be seen within bundles. I.e. bundles span across the different regions and their content changes accordingly. We don't know what causes these two different outcomes.
